# Carbapenem-resistant *Enterobacteriaceae* in the livestock, humans and environmental samples around the globe: a systematic review and meta-analysis

**DOI:** 10.1038/s41598-024-64992-8

**Published:** 2024-07-15

**Authors:** Barbra Tuhamize, Joel Bazira

**Affiliations:** Department of Microbiology, Faculty of Medicine, Mbarara University of Science and Technology, Mbarara, Uganda

**Keywords:** *Enterobacteriaceae*, Carbapenem-resistance, Resistance genes, Microbiology, Molecular biology

## Abstract

Carbapenem-resistant *Enterobacteriaceae* (CRE) have diminished treatment options causing serious morbidities and mortalities. This systematic review and meta-analysis assessed the prevalence and associated factors of *Enterobacteriaceae* infections in clinical, livestock and environmental settings globally. The population intervention comparison and outcome strategy was used to enroll studies using the preferred reporting system for systematic review and meta-analysis to include only cross-sectional studies. Search engines used to retrieve articles included journal author name estimator, PubMed, Google Scholar and African Journals Online (AJOL). The Newcastle–Ottawa scale was used to assess the quality of studies. Sixteen articles from 2013 to 2023 in Africa, Asia, Europe and South America were studied. The pooled prevalence of CRE was 43.06% (95% CI 21.57–66.03). *Klebsiella pneumoniae* (49.40%), *Escherichia coli* (26.42%), and *Enterobacter cloacae* (14.24%) were predominant. *Klebsiella pneumoniae* had the highest resistance with the *bla*KPC-2 in addition to *bla*NDM, *bla*OXA-48, *bla*IMP and *bla*VIM. The *bla*KPC-2 genes occurrence was associated with environmental (*P*-value < 0.0001) and South American studies (*P*-value < 0.0001), but there was no difference in the trends over time (*P*-value = 0.745). This study highlights the high rates of CRE infections, particularly within *bla*KPC production. Monitoring and surveillance programs, research and infection control measures should be strengthened. Additionally, further studies are needed to explore the mechanisms driving the predominance of specific bacterial species and the distribution of resistance genes within this bacterial family.

## Introduction

*Enterobacteriaceae* are a large and diverse group of gram-negative bacteria that are found in the environment and in the gut of animals, birds and humans. They can cause a variety of infections, including urinary tract infections, pneumonia, and sepsis. In recent years, there has been an increasing prevalence of *Enterobacteriaceae* that are resistant to several antibiotics^1^.

Carbapenem-resistant *Enterobacteriaceae* (CRE) are a group of Gram-negative bacteria that have become resistant to carbapenems, which are a class of antibiotics that are last-line treatments for many serious infections. The CRE can be found in a variety of settings, including hospitals, nursing homes and several other healthcare facilities^1^. However, they are also increasingly being found in the environment^2–4^ and among people who have or not been recently hospitalized and livestock^5–8^. The emergence of CRE in these various settings, has raised concerns about the widespread dissemination of these multidrug-resistant pathogens^9,10^. Various carbapenamases have been detected in *Enterobacteriaceae* organisms, categorized into three classes of β-lactamases: Ambler class-A (KPC, SME, IMI, NMC, GES types), class-B metallo-ß-lactamases (VIM, IMP, GIM, SMP, and NDM types), and class-D β-lactamases (primarily OXA-48). Additionally, there are rare chromosome-encoded class C enzymes. These enzyme groups all provide resistance to broad-spectrum antibiotics, including carbapenems^11^. Transfer of these resistance genes facilitated by processes such as transformation, transduction, and conjugation, is a common occurrence in Carbapenem-Resistant *Enterobacteriaceae* (CRE)^11^.

Several genes, including *bla*OXA-58, *bla*OXA-48, *bla*OXA-23, *bla*VIM-2, and *bla*VIM-4, were identified as playing a role in carbapenem resistance^12^. These genes were found to be commonly present in carbapenemase-producing bacteria during outbreaks in Africa by 2010^12^. Carbapenemase-encoding genes, *bla*VIM, *bla*OXA, *bla*IMP, *bla*KPC, and *bla*NDM, showed variable distribution patterns among clinical isolates collected from Mulago National Referral Hospital in Uganda^13^. Additionally, bacterial strains producing Carbapenemase and carrying *bla*VIM and *bla*OXA-48 genes were also detected in specimens from patients at Mbarara Regional Referral Hospital^14^.

Elsewhere, these organisms have also been found in animals raised for food due to the excessive use of antibiotics in animal farming. The presence of CRE in livestock raises concerns about the potential transmission of antibiotic-resistant bacteria to humans. If these bacteria are able to move from animals to humans, it creates a significant risk to public health by reducing the effectiveness of treatment for severe infections^15,16^. The detection of CRE in both human and animal samples indicates that the environmental sources in rural southwestern Uganda could be aiding in the dissemination of resistant bacteria^17^. Therefore, it is essential to identify these sources and enforce necessary control measures^17^.

Therefore, there is a need for collective efforts globally to control AMR including CRE. The One Health concept, which emphasizes the interconnectedness of human, animal, and environmental health, is crucial for tackling the global issue of CRE prevalence. Collaborative efforts across these domains are essential for comprehensive control measures. Understanding the genetic constitution of the CRE would serve the primary role of unlocking this burden.

### Aim of the study

The aim of this systematic review and meta-analysis was to assess the prevalence and transmission dynamics of *Enterobacteriaceae* in clinical, livestock, and environmental settings globally.

## Methods

### Selection

The population intervention comparison and outcome (PICO) strategy was used to make selection of the studies. Only cross-sectional studies that employed the molecular assays to assess CRE during data collection were included in the review. They should have been published in English. For clinical studies, those that included stool specimens were excluded in the review. Comparative groups were clinical, environmental and livestock with prevalence as the measure of outcome.

### Data search

**Figure 1:**
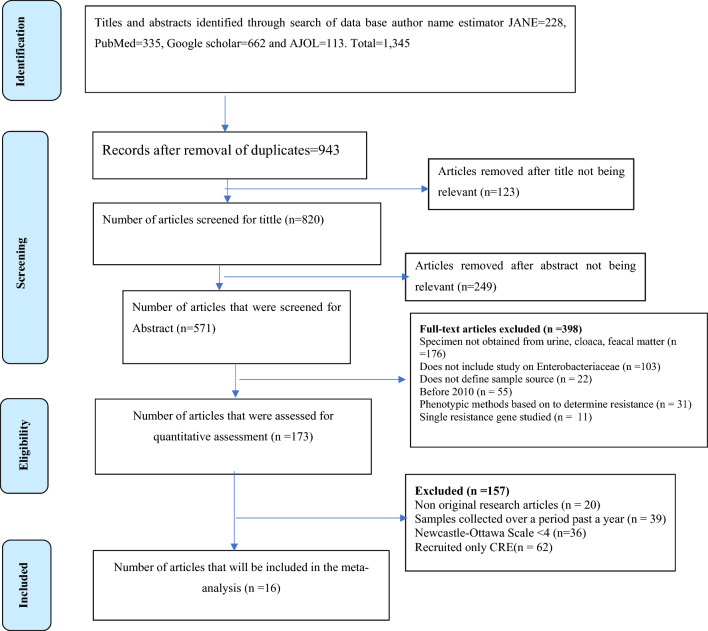
Journal selection for inclusion in the meta-analysis.

The Preferred Reporting System for Systematic Review and Meta-analysis (PRISMA) standards was used to guide the process that includes literature search and data extraction, data cleaning, data analysis and interpreting result a seen in Fig. 1. The primary studies that investigated epidemiology, transmission dynamics of molecular analysis of carbapenem resistant *Enterobacteriaceae* in community and health care settings were adopted for the systematic review. Search engines used to retrieve the articles included journal author name estimator (JANE), PubMed, Google scholar and African Journals Online (AJOL). Data from articles published between January 2010 and December 2022 was included.

Search terms were (“Carbapenem Resistant *Enterobacteriaceae*”), (“Hospital Carbapenem Resistant *Enterobacteriaceae*”), (“Community Carbapenem Resistant *Enterobacteriaceae*”), (“Carbapenem Resistant *Enterobacteriaceae* in livestock”), (“Environment Carbapenem Resistant *Enterobacteriaceae*”) and, from the articles databases. The (“Phylogeny relationship of Carbapenem Resistant *Enterobacteriaceae*”) and (“Nucleotide homology comparison of Carbapenem Resistant *Enterobacteriaceae*”) search term were used to extract data from the DNA databases. Snow balling was done after text review of references for related articles. Search articles were organized in Endnote from which the duplicates were removed.

### Screening

A thorough screening of the articles was performed starting with title and abstract and then full text reading. This was performed by two reviewers (Ms. Barbra Tuhamize and Assoc Prof. Joel Bazira) from 1st August to 21st October 2023. The two also performed quality appraisal of the recruited articles as cross-sectional in nature to avoid bias using the Cochrane risk of bias assessment tool. Any disagreement between the two reviewers were settled by a consensus during the weekly evaluation meetings that sat every Wednesday of the week.

### Assessment of data quality

The Newcastle–Ottawa scale (NOS) was used to assess the quality of studies that passed the text reviews. Studies with a NOS of three or less were dropped from the meta-analysis.

### The meta-analysis (quantitative data synthesis)

The effect size for each study was checked and later the combined estimate of effect size generated. The random effects model was considered to include heterogeneity. The forest plot was drawn to check the heterogeneity. The funnel plot and the Egger’s and Beggs tests were used to assess the publication bias. Sensitivity analysis was performed by removing one article at ago and check result variation.

Subgroup analysis was later performed for the highly predominant *bla*KPC gene amongst the different sample sources, continent and trends over time. The distribution of the *bla*KPC gene among the sample sources and the different continents was performed using the metanalysis of pooled prevalence using the chi square test at 95% CI and trends over time from 2013 to 2023 using linear regression at 95% CI.

### Ethics approval and consent to participate

This is a systematic review and meta-analysis. We reviewed published studies and only those that had received ethics approval during data collection.

## Results

### Systematic review

The search and systematic review yielded 16 studies from 2013 to 2023 in Africa, Asia, Europe and South America (Table 1) using the PRISMA standards guide (Fig. 1).

**Table 1:** General characteristics of Characteristics of studies included in the review.

SN	First Author	Publication year	Country	Continent	Sample source	Sample type	Sample size	Organism	Genes per bacteria	Individual genes					
										blaNDM-1	blaNDM-5	blaIMP-4	blaKPC-2	blaOXA-48	blaVIM
1	Makharita et al.^5^	2022	Egypt	Africa	Human	Urine	37	*Klebsiella pneumoniae* (18)	*bla*KPC-2 (9) & *bla*GES (9)				9		
								*Escherichia coli*	*bla*KPC-2 (1) & *bla*GES (1)				1		
								*Enterobacter aerogenes* (2)	0						
								*Proteus mirabilis* (1)	0						
2	Ribeiro et al.^18^	2013	Brazil	South America	Human	Urine	345	*Klebsiella pneumoniae* (93)	*bla*KPC-2 (5)				5		
								*Klebsiella oxytoca* (1)	0						
								*Enterobacter aerogenes* (31)	0						
								*Enterobacter cloacae* (193)	*bla*KPC-2 (6)				6		
								*Escherichia coli* (18)	0						
								*Proteus mirabilis* (1)	0						
								*Serratia marcescens* (8)	*bla*KPC-2 (3)				3		
3	Nabti et al.^6^	2021	Algeria	North Africa	Human	Urine	123	*Klebsiella pneumoniae* (52)	*bla*OXA-48 (4)					4	
								*Escherichia coli* (24)	*bla*OXA-48 (1)					1	
								*Enterobacter cloacae* (19)	*bla*NDM-1 (1)	1					
								*Proteus mirabilis* (11)	0						
								*Proteus vulgaris* (1)	0						
								*Serratia marcescens* (4)	0						
								*Citrobacter freundii* (4)	0						
								*Citrobacter braakii* (2)	0						
								*Providencia rettgeri* (1)	0						
4	Cizmeci et al.^7^	2017	Turkey	Europe	Human	Urine	76	*Klebsiella pneumoniae* (69)	*bla*NDM-1 (15), *bla*OXA-48 (50) & *bla*VIM (2)	15				50	2
								*Enterobacter cloacae* (4)	*bla*NDM-1 (3) & *bla*VIM (1)	3					1
								*Enterobacter aerogenes* (4)	*bla*OXA-48 (2)					2	
								*Escherichia coli* (1)	*bla*OXA-48 (1)					1	
5	Li et al.^19^	2018	China	Asia	Human	Urine	302	*Escherichia coli* (34)	*bla*NDM-5 (18) & *bla*KPC-2 (4)		18		4		
								*Klebsiella pneumoniae* (268)	*bla*NDM-5 (12), *bla*IMP-4 (4) & *bla*KPC-2 (237)		12	4	237		
6	Hamza et al.^41^	2016	Egypt	Africa	Bird	faecal	100	*Klebsiella pneumoniae* (35)	*bla*NDM-5 (15), *bla*KPC-2 (11) & *bla*OXA-48 (11)		15		11	11	
7	Jin et al.^1^	2016	China	Asia	Environment	Sewage	37	*Klebsiella pneumoniae* (11)	*bla*KPC-2 (4)				4		
								*Escherichia coli (10)*							
								*Enterobacter cloacae* (13)	*bla*NDM-1 (1) & *bla*KPC-2 (5)	1			5		
								*Citrobacter freundii* (3)	*bla*NDM-1 (2)	2					
8	Montezzi et al.^2^	2015	Brazil	South America	Environment	recreational water	18	*kluyvera* (2)	*bla*KPC-2 (2)				2		
								*Enterobacter cloacae* (8)	*bla*KPC-2 (8)				8		
								*Citrobacter freundii* (1)	*bla*KPC-2 (1)				1		
								*Klebsiella pneumoniae* (1)							
9	Solgi et al.^20^	2017	Iran	Asia			95	*Escherichia coli* (43)				13			
								*Enterobacter cloacae* (12							
10	Sonnevend et al.^21^	2015	Saudi Arabia	Asia	Human	Urine	265	*Klebsiella pneumoniae* (145)		85				50	6
								*Klebsiella oxytoca* (2)	2						
								*Escherichia coli* (28)		9				9	1
								*Enterobacter cloacae* (17)		2				8	5
								*Serratia marcescens* (1)							
								Citrobacter freundii (3)							
								*Citrobacter koseri* (1)							
								*Providencia stuartii* (1)							
								*Morganella morganii* (2)							
11	Okoche et al.^13^	2015	Uganda	Africa	Human	Urine	192	*Klebsiella pneumoniae* (78)		4		5	3	7	16
								*Klebsiella oxytoca* (4)	4					1	
								*Escherichia coli* (84)				6	4	8	1
								*Enterobacter cloacae* (9)		1			1	2	1
								*Proteus mirabilis* (6)			1		1		
								*Proteus vulgaris* (3)							
								*Citrobacter freundii* (8)							1
12	Su et al.^3^	2023	China	Asia	Bird	environmental	707	*Klebsiella oxytoca* (50)	50	5				3	
								*Escherichia coli* (168)		6				3	
								*Proteus mirabilis* (33)						1	
13	Alraddadi et al.^42^	2022	Saudi Arabia	Asia	Human	Urine	189	*Klebsiella pneumoniae* (165)	*bla*NDM-1 (20), *bla*KPC-2 (1) & *bla*OXA-48 (10)	20			1	10	
14	Feng et al.^8^	2021	China	Asia	Animal	meat	760	*Escherichia coli* (24)		4				5	
								*Klebsiella pneumoniae* (9)		3			5		
								*Escherichia coli* (43)		39					
								Enterobacter cloacae (1)							
15	Abdallah et al.^10^	2015	Egypt	Africa	Bird	meat	112	*Klebsiella pneumoniae* (44)	*bla*NDM-1 (11)	11					
								*Klebsiella oxytoca* (2)	*bla*NDM-1 (1)	1					
								*Escherichia coli* (38)							
								*Enterobacter aerogenes* (4)							
								*Enterobacter cloacae* (17)							
								*Citrobacter freundii* (1)							
16	Piedra-Carrasco et al.^4^	2017	Spain	Europe	Environment	water	224	*Klebsiella pneumoniae* (1)	*bla*KPC-2 (1)				1		
								*Klebsiella oxytoca* (1)	*bla*KPC-2 (1)				1		
								*Escherichia coli* (3)	*bla*KPC-2 (3)				3		
								*Enterobacter cloacae* (2)	*bla*KPC-2 (2) & *bla*VIM (2)				2		2
								*Raoultella ornithinolytica* (1)	*bla*VIM (1)						1

These 16 studies that were included in the study, were caried out from African, Asia, Europe and South America using samples from environmental, livestock and clinical specimens^1–10,13,18–21^. Several *Enterobacteriaceae* were reported to carry resistance genes as shown in (Table 1) below.

**Figure 2:**
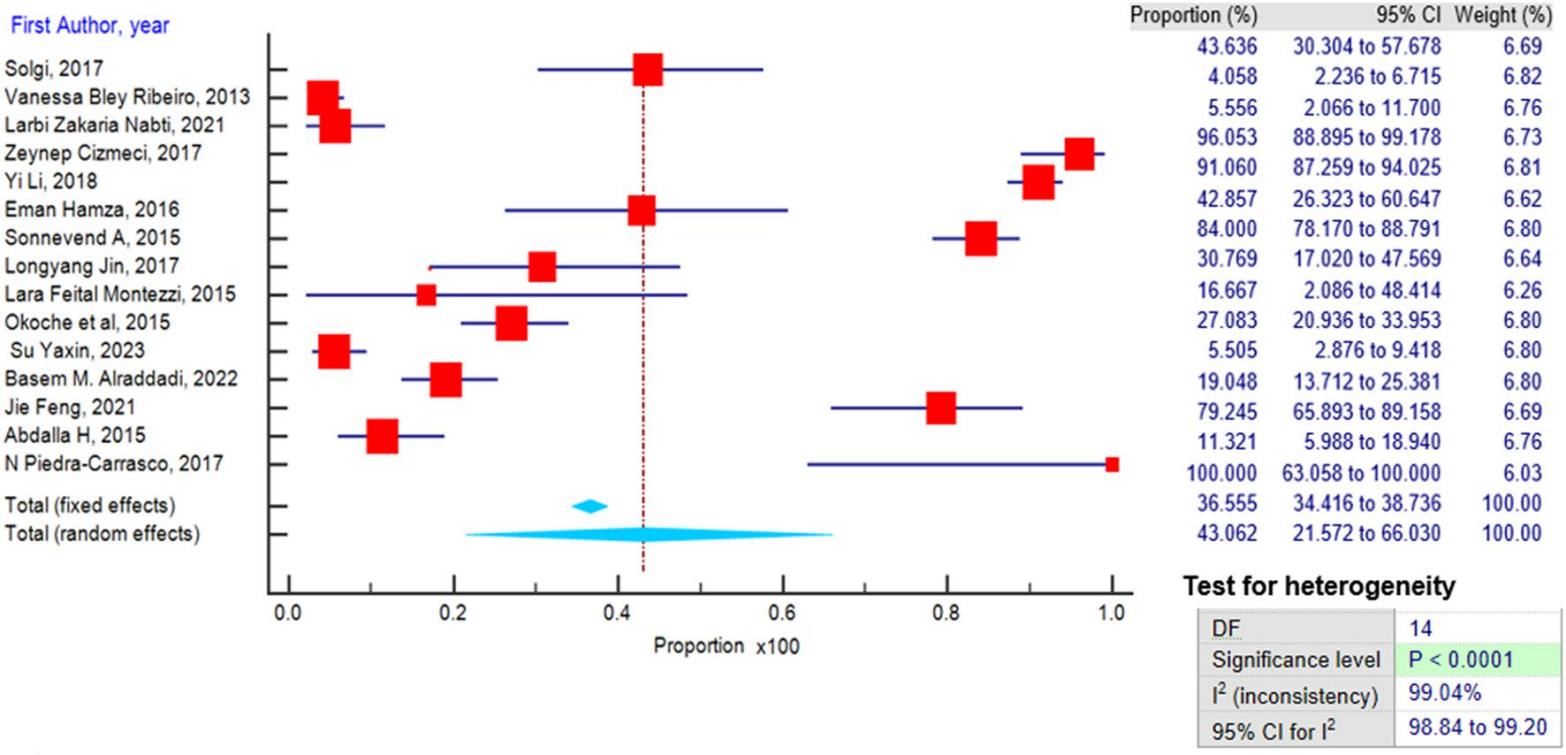
Forest plot presenting the prevalence of CRE and distribution.

**Figure 3:**
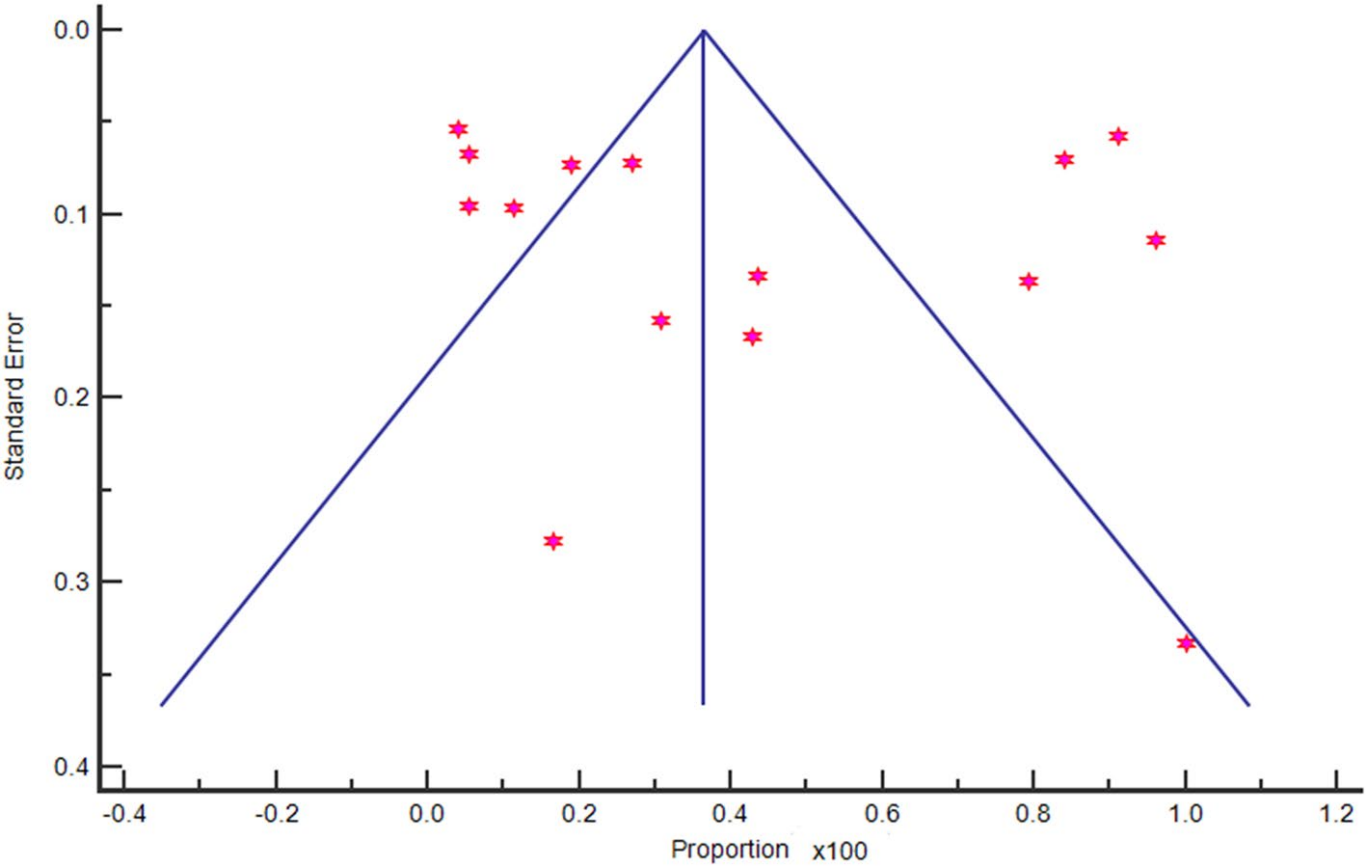
Funnel plot showing the heterogeneity index for the study results.

A meta-analysis was conducted among the 16 articles. The pooled prevalence of CRE was 43.06% (95% confidence interval [CI] 21.57–66.03) using a random effect model, catering for heterogeneity (Fig. 2). Bias was assessed using the funnel plot indicated in (Fig. 3) below.

The predominant bacteria species were, *Klebsiella pneumoniae* (49.40%), *Escherichia coli* (26.42%), and *Enterobacter cloacae* (14.24%). Other bacteria species included *Klebsiella oxytoca* (2.99%), *Proteus mirabilis* (2.55%), *Enterobacter aerogenes* (1.95%), *Proteus vulgaris* (0.19%), *Kluyvera* (0.19%), *Morganella morganii* (0.19%), *Citrobacter freundii* (0.99%), *Serratia marcescens* (0.65%), *Citrobacter braakii* (0.19%), and *Citrobacter koseri* (0.05%), *Providencia rettgeri* (0.05%), *Providencia stuartii* (0.05%), *Raoultella ornithinolytica* (0.05%) were the least isolated (Table 2).

**Table 2:** The common of bacteria species isolated.

SN	First Author	Sample size	Total *Enterobacteriaceae*	CRE positive	*Klebsiella pneumoniae*	*Klebsiella oxytoca*	*Escherichia coli*	*Enterobacter aerogenes*	*Enterobacter cloacae*	*Serratia marcescens*	*Proteus mirabilis*	*Proteus vulgaris*	*Citrobacter freundii*	*kluyvera*	*Raoultella ornithinolytica*	*Morganella morganii*
1	Solgi et al.^20^	95	55	24			43		12							
2	Makharita et al.^5^	37	31	11	18		11	2								
3	Ribeiro et al.^18^	345	345	14	93	1	18	31	193	8	1					
4	Nabti et al.^6^	123	108	6	52		24		9	4	11	1	4			
5	Cizmeci et al.^7^	76	76	73	69		1	2	4							
6	Li et al.^19^	302	302	275	268		34									
7	Hamza et al.^41^	100	35	15	35											
8	Sonnevend et al.^21^	265	200	168	145	2	28		17	1			3			2
9	Jin et al.^1^	39	39	12	11		10		13				3	2		
10	Montezzi et al.^2^	18	12	2	1				8				1	2		
11	Okoche et al.^13^	192	192	52	78	4	84		9		6	3	8			
12	Su et al.^3^	707	218	12		50	168				33					
13	Alraddadi et al.^42^	189	189	36	165		24									
14	Feng et al.^8^	760	53	42	9		43		1							
15	Abdallah et al.^10^	112	106	12	44	2	38	4	17				1			
16	Piedra-Carrasco et al.^4^	224	8	8	1	1	3		2						1	

High prevalence of CRE was exhibited among *Klebsiella pneumoniae* isolates. The gene occurrence was higher for the *bla*KPC-2, followed by *bla*NDM, *bla*OXA-48, *bla*IMP, *bla*VIM, and the least was *bla*GES, no resistance was reported by *Citrobacter braakii*, *Klebsiella oxytoca*, *Proteus mirabilis*, *Proteus vulgaris*, and *Providencia rettgeri* (Table 3). Organisms carried resistance genes from one to as many as seven per bacteria.

**Table 3:** CRE gene distribution for the different *Enterobacteriaceae*.

Organism	Number of bacteria	Genes carried						Total genes
		*bla*KPC-2	*bla*OXA-48	*bla*NDM-5	*bla*VIM	*bla*NDM-1	*bla*IMP-4	
*Klebsiella pneumoniae*	989	582	313	106	58	16	7	1082
*Escherichia coli*	529	5	2	18				25
*Enterobacter cloacae*	285	14			1	4		19
Others	119	6	2	0	0	1	0	10
Total		607	317	124	59	21	7	1136

**Figure 4:**
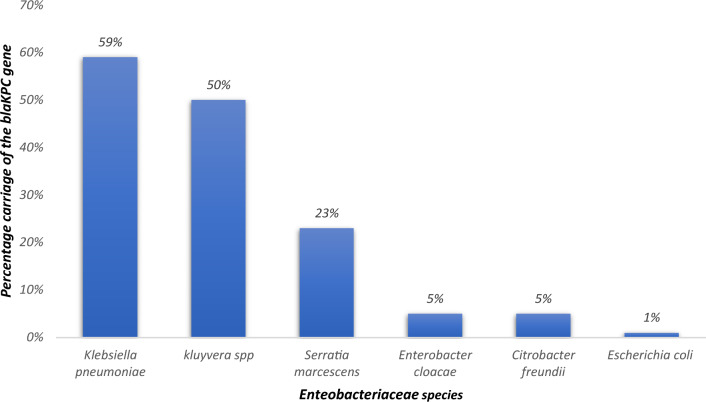
Comparison of the blaKPC gene occurrence among the Bacteria species.

The *bla*KPC-2 gene was dominant, carried more among *Klebsiella pneumoniae* (582/989; 59%), followed by *Kluyvera spp* (2/4; 50%), *Serratia marcescens* (3/13; 23%) and lowly carried by *Enterobacter cloacae* (14/285; 5%), *Citrobacter freundii* (1/20; 5%) and *Escherichia coli* (5/529; 1%) (Fig. 4).

**Figure 5:**
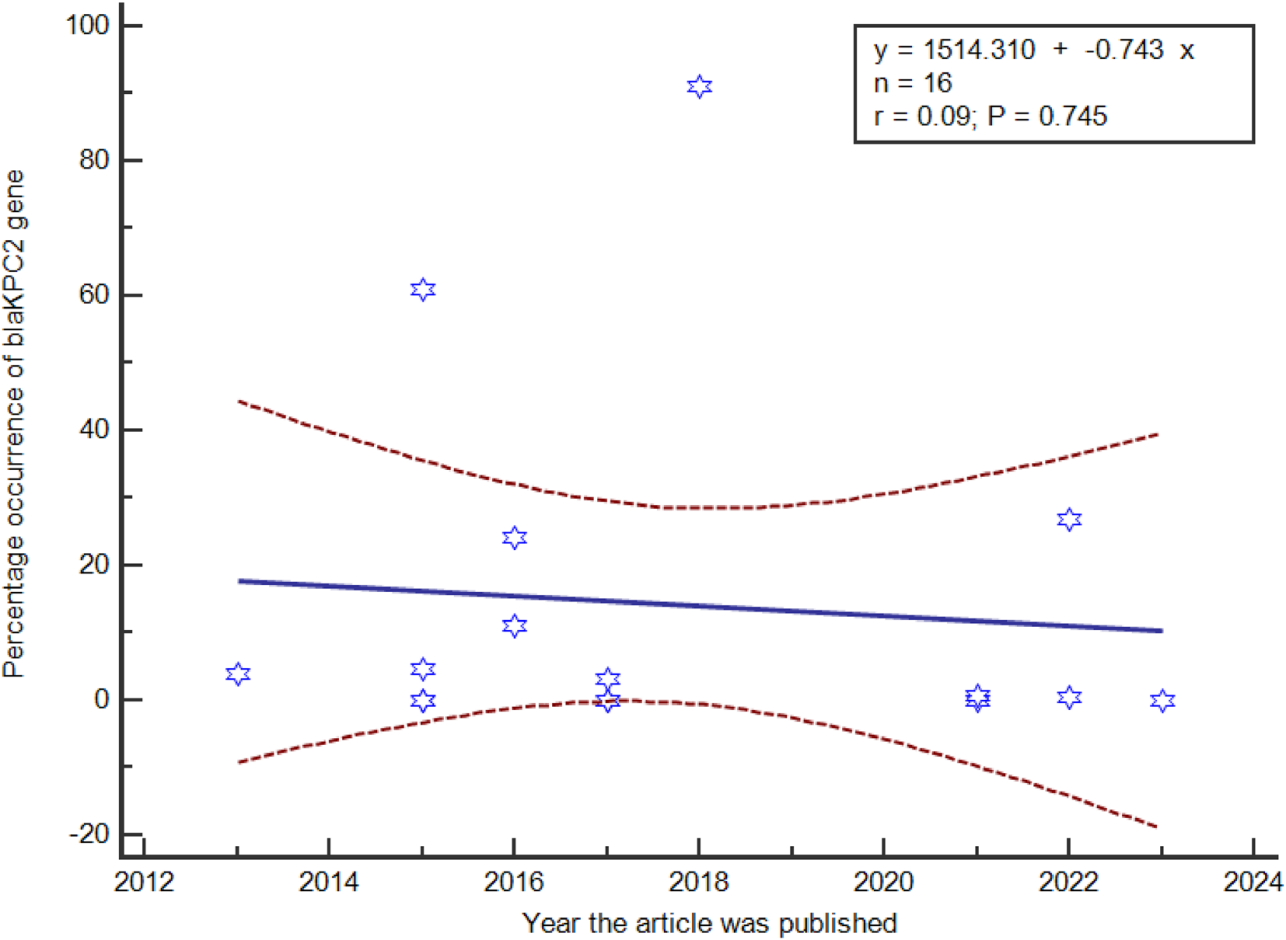
blaKPC gene occurrence over time (2013 to 2023).

The highly prevalent *blaKPC* gene’s occurrence was evaluated amongst the sample sources (Table 4), continents (Table 5) and trends over time from 2013 to 2023 (Fig. 5). Environmental samples recorded the highest prevalence of the *blaKPC* gene compared the humans (*P*-value < 0.0001) and livestock (*P*-value < 0.0001). Amongst the continents, South American studies produced the highest as compared to Asia (*P*-value < 0.0001), Africa (*P*-value < 0.0001) and Europe (*P*-value < 0.0001). There was no significant difference in the trends of occurrence of the *blaKPC* gene over time (*P*-value = 0.745).

**Table 4:** The occurrence of *bla*KPC gene among the sample sources for bacteria.

Continent	Number of records	Analysis of the prevalence	*p* Value	Analysis of heterogeneity	p. het	Model
		pooled prevalence (95%CI)		I^2^ (95% CI)		
Environmental	3	24.786 (1.627 to 62.751) ref		95.63% (90.45 to 98.00)	< 0.0001	Random
Human	9	7.63 (0.155 to 31.797)	< 0.0001	99.40 (99.26 to 99.51)	< 0.0001	Random
Livestock	4	1.335 (0.0330 to 4.488)	< 0.0001	92.25% (83.36 to 96.39)	< 0.0001	Random

**Table 5:** Comparison of the blaKPC gene occurrence among the continents.

Continent	Number of records	Analysis of the prevalence	*p* Value	Analysis of heterogeneity	p. het	Model
		pooled prevalence (95%CI)		I^2^ (95% CI)		
South America	2	26.416 (1.725 to 87.626) ref		97.05% (92.32 to 98.86)	< 0.0001	Random
Asia	7	8.39 (0.263 to 35.570)	< 0.0001	99.62% (99.53 to 99.69)	< 0.0001	Random
Africa	5	5.363 (0.693 to 14.041)	< 0.0001	92.03% (84.38 to 95.93)	< 0.0001	Random
Europe	2	1.649 (0.00359 to 6.194)	< 0.0001	72.72% (0.00 to 93.86)	0.0555	Random

## Discussion

The findings of this systematic review and meta-analysis provide critical insights into the prevalence and characteristics of *Enterobacteriaceae* infections, as well as the distribution of carbapenemase resistance genes within this bacterial family. These bacteria can cause a wide range of infections, including urinary tract infections, surgical site infections, pneumonia, bloodstream infections, and diarrhea^22–24^. *Enterobacteriaceae* infections can be particularly serious in young children, the elderly, and people with weakened immune systems^23^. The results from this systematic review and meta-analysis have important implications for public health to curb AMR.

### Prevalence of *Enterobacteriaceae* infections

This systematic review and meta-analysis were unique that they pooled studies that researched on several sample sources including humans (urine samples), livestock (feces and cloaca) and in the environment.

The findings of this meta-analysis, based on a comprehensive review of 16 articles, provide important insights into the global prevalence of Carbapenem-resistant *Enterobacteriaceae* infections. The pooled prevalence of CRE infections was estimated at 43.06%, (95% CI 21.57–66.03%). Notably, a random effect model was employed to account for the observed heterogeneity among the studies. This finding is consistent with the increasing reports of CRE infections from various parts of the world, reflecting the growing challenge of antibiotic resistance in clinical and non-clinical settings^25^. The observed pooled prevalence of 43.06% underscores the alarming global prevalence of CRE infections, suggesting that this issue is more widespread than previously thought.

The wide 95% confidence interval (21.57–66.03%) reflects the substantial variability in CRE prevalence across different regions and populations. The heterogeneity observed in the meta-analysis may be attributed to several factors, including variations in healthcare practices, antibiotic use, and infection control measures among different countries and healthcare settings^26^. This heterogeneity highlights the need for a multifaceted approach to address CRE, as a one-size-fits-all strategy may not be sufficient.

It is essential to contextualize the implications of this prevalence estimate in the broader landscape of antimicrobial resistance. CRE is particularly concerning due to their resistance to carbapenem antibiotics, which are often considered the last line of defense against severe infections caused by Gram-negative bacteria^24^. The high prevalence of CRE signifies a critical need for effective interventions to curb the spread of resistant strains and preserve the efficacy of existing antibiotics. Like Kelly et al.^25^ suggested, this is one area prime to support especially through the one health approach if AMR must be tamed.

In addition to the overall prevalence estimate, the findings of this meta-analysis provide a foundation for understanding the scope of the CRE problem across various populations and regions. This information is instrumental in guiding public health policies, infection control strategies, and antimicrobial stewardship programs aimed at mitigating the impact of CRE.

To address the challenges posed by CRE, we concur with^27^ that a comprehensive approach is warranted, including the judicious use of antibiotics, stringent infection control measures in healthcare facilities, and active surveillance to detect and contain CRE outbreaks. Furthermore, international collaboration is essential to share best practices and prevent the further dissemination of CRE strains.

It is important to note that the limitations of this meta-analysis include potential publication bias and variations in data reporting and methodology among the included studies. Future research should continue to monitor CRE prevalence and investigate the genetic mechanisms underlying carbapenem resistance to develop targeted interventions.

### Predominant bacterial species

The identification of predominant bacterial species within the *Enterobacteriaceae* family is essential for understanding the epidemiology of these infections. *Klebsiella pneumoniae* emerged as the most prevalent species, accounting for 49.40% of isolates followed by *Escherichia coli* and *Enterobacter cloacae* with prevalence rates of 26.42% and 14.24%, respectively.

The predominance of *K. pneumoniae* would be explained by their isolation from clinical studies abundantly, whose (the clinical samples) sample sizes were higher than those from the environment and livestock. This distribution of predominant species suggests that certain *Enterobacteriaceae* members may possess specific virulence factors or resistance mechanisms that contribute to their prevalence. Some known highly virulent bacteria including *Salmonella* and *Shigella* were not reported, but this is explained by them being enteric (by transmission) and very rarely or occassionally cause urinary tract infections^28^.

The abundance of *Enterobacteriaceae* in environmental sources is an indicator of their animal and human fecal sources^29^, and the cross distribution of carbapenem resistance genes worries AMR control program and the One health approach^2,30,31^. Moreover, *Kluyvera*, a less human pathogen (reference) was found to carry *bla*KPC genes (that is predominantly isolated from clinical specimens). This highlights a need to study comprehensively several other environmental sources such as indoor air and surface areas from public places including schools, hotels, office spaces and day care centers among others.

Further research is warranted to elucidate the factors driving the predominance of these species and to develop targeted interventions for their control.

### Carbapenemase resistance genes

One of the most concerning aspects of *Enterobacteriaceae* infections is the emergence and spread of carbapenem-resistant strains (CRE). Our analysis identified *Klebsiella pneumoniae* as a major contributor to CRE prevalence, with a notably high occurrence of carbapenemase resistance genes within this species.

Among the carbapenemase resistance genes, *bla*KPC-2 exhibited the highest occurrence, followed by *bla*NDM, *bla*OXA-48, *bla*IMP, and *bla*VIM being the least prevalent. The diversity of carbapenemase genes highlights the complexity of antimicrobial resistance within the *Enterobacteriaceae* family. In environmental and sewage sources, several authors have reported about the abundance of the CRE and their potential to dissemination^8,32–35^. Therefore, the presence of these genes underscores the urgent need for stringent infection control measures and the judicious use of antibiotics to prevent further dissemination of CRE^33^.

It is worth noting that certain bacterial species, including *Proteus mirabilis (51)* and *Klebsiella oxytoca (60)* never carried any resistance genes. These species could serve as valuable models for studying resistance mechanisms and potentially inform strategies for combating CRE. However, a similar observation cannot be made for *Raoultella ornithinolytica, Proteus vulgaris, Citrobacter braakii, Citrobacter koseri, Providencia rettgeri, Providencia stuartii* and *Morganella morganii* for not expressing any resistance genes because their frequencies were too low to generate any statistical inference.

### The *bla*KPC dominance

The findings of this systematic review and meta-analysis reveal crucial insights into the prevalence and distribution of the *bla*KPC-2 gene among different bacterial species in diverse settings, shedding light on the genetic determinants of Carbapenem-resistant *Enterobacteriaceae* (CRE).

The dominance of the *bla*KPC-2 gene, particularly in *Klebsiella pneumoniae*, is a significant and concerning finding. *Klebsiella pneumoniae* is a well-known nosocomial pathogen responsible for a wide range of infections, and its association with the *bla*KPC-2 gene indicates the potential for challenging therapeutic outcomes^36^. The high prevalence of this gene among *Klebsiella pneumoniae* (59%) is similar to what^37^ reported in China. This underscores the importance of this species as a key contributor to the CRE problem, especially in healthcare settings where carbapenems are frequently used.

Furthermore, the detection of the *bla*KPC-2 gene in other species like *Kluyvera* spp, *Serratia marcescens, Enterobacter cloacae, Citrobacter freundii*, and *Escherichia coli* highlights the diversity of bacterial species capable of carrying this resistance gene. While these species might not be as common as *Klebsiella pneumoniae* in clinical infections, their capacity to harbor *bla*KPC-2 is a critical concern. In particular, the presence of this gene in *Kluyvera* spp and *Serratia marcescens* (which are commonly found in the environment) is noteworthy, given the potential for these species to act as reservoirs for resistance genes and share them with more clinically relevant pathogens.

The variation in the prevalence of the *bla*KPC-2 gene across different bacterial species suggests that the distribution of carbapenem resistance determinants is not uniform and may be influenced by species-specific factors and selective pressures. For example, *Enterobacter cloacae* and *Escherichia coli* exhibit lower prevalences (5% and 1%, respectively) of the *bla*KPC-2 gene, indicating that other resistance mechanisms may be more prevalent in these species or that their association with CRE may be mediated by different resistance genes encoded by plasmids or bacteriophages^38^. These variations are consistent with previous studies that have reported differential resistance gene carriage among different *Enterobacteriaceae* species^39^.

The identification and prevalence of specific carbapenemase genes, such as the *bla*KPC-2 gene, within CRE are critical for understanding the dynamics of resistance gene dissemination across different reservoirs, including environmental samples, humans, and livestock. This systematic review and meta-analysis have revealed noteworthy findings regarding the dominance and prevalence of the *bla*KPC-2 gene in these diverse reservoirs.

Understanding the diversity of species carrying the *bla*KPC-2 gene is crucial for the development of effective infection control and antimicrobial stewardship strategies. Also, to focusing on well-known CRE pathogens like *Klebsiella pneumoniae*, efforts should also target species with lower prevalence but potential for gene dissemination. Additionally, monitoring and surveillance programs should consider the presence of the *bla*KPC-2 gene in environmental samples, as these reservoirs can contribute to the spread of resistance determinants among both clinical and non-clinical settings.

The findings of this study emphasize the importance of continued research into the genetic determinants of CRE and the factors influencing their distribution among different bacterial species. By understanding the prevalence of resistance genes like *bla*KPC-2, we can better address the evolving threat of CRE and develop strategies to mitigate its impact on public health.

The study by^3^ only reported *bla*NMD and *bla*OXA but not *bla*IMP, *bla*KPC, *bla*VIM and *bla*GES similar to. This was different from other studies that reported at least one of these genes with dominance of *bla*KPC.

### Attributes to *bla*KPC occurrence

#### The ecological niche of the *blaKPC* gene

In this research, a higher prevalence of the *bla*KPC gene in environmental samples was obtained compared to both humans and livestock. This result suggests that environmental sources, such as water, soil, or wastewater, may serve as a reservoir for CRE carrying the *bla*KPC gene especially in community transmission. Several factors could contribute to this higher prevalence in environmental though none of the studies assessed these factors. The environment can act as a bridge for CRE transmission between humans, animals, and the surrounding ecosystem^1^. The contamination of the environment with CRE from human or livestock sources can establish a cycle of transmission.

Understanding the higher prevalence of the *bla*KPC gene in environmental samples emphasizes the need for improved environmental surveillance and management to reduce the dissemination of carbapenem resistance. It is essential to consider the complex interplay between human activities, the environment, and antimicrobial resistance.

These findings underscore the importance of adopting a “One Health” approach, which recognizes the interconnectedness of human, animal, and environmental health. Efforts should be made to monitor and mitigate CRE in all these domains to effectively combat the global spread of antibiotic resistance.

The systematic review and meta-analysis of CRE prevalence and genetic determinants, particularly the *bla*KPC-2 gene, have yielded intriguing insights into the distribution of carbapenem resistance across continents. The findings not only highlight the dominance of the *bla*KPC-2 gene but also reveal significant variations in its prevalence among different continents.

#### Variation in prevalence among continents

The marked differences in *bla*KPC gene prevalence among continents are of substantial concern and indicate significant geographical disparities in CRE distribution:

The highest prevalence of the *bla*KPC gene in South American studies, as compared to Asia, Africa, and Europe, points to a particularly critical situation in this region. South America has witnessed increasing challenges in healthcare-associated infections and antimicrobial resistance, which may be contributing to the high prevalence of CRE.

Asia came second to South America and this is similar to recent reports. Asia has been a significant focus of CRE research due to its large population and diverse healthcare systems. Various studies have highlighted the presence of *bla*KPC-carrying CRE in the region, indicating the multifaceted nature of the issue.

The comparatively lower prevalence of the *bla*KPC gene in Africa may be influenced by variations in healthcare infrastructure, antimicrobial use, and epidemiological factors. However, CRE has been a growing concern in parts of Africa, and its prevalence is expected to evolve.

Europe's comparatively lower prevalence suggests a more controlled situation, likely due to stringent infection control measures and antibiotic stewardship programs in place. However, localized outbreaks and variations within European countries are also evident.

#### Consistent trends in *bla*KPC gene occurrence over time

The systematic review and meta-analysis findings, highlighting the dominance of the *bla*KPC-2 gene, and the consistent trends in the occurrence of the *bla*KPC gene over a ten-year period (2013–2023), provide valuable insights into the dynamics of carbapenem resistance in CRE. These findings indicate the persistence and resilience of this carbapenemase gene within the global CRE landscape.

It is important to note that while the overall trends in the occurrence of the *bla*KPC gene appear stable, there may be localized variations and fluctuations within different regions, healthcare facilities, and ecological niches. These nuances could be influenced by factors such as local infection control practices, antibiotic use patterns, and the introduction of new CRE strains^25,40^. The observed stability in the occurrence of the *bla*KPC gene from 2013 to 2023 reflects the ongoing challenges posed by carbapenem resistance. These results emphasize the importance of continued vigilance, surveillance, and the development of strategies to combat CRE and its associated carbapenemase genes.

### Limitations

While this systematic review and meta-analysis provide valuable insights, some limitations should be acknowledged. There may be heterogeneity among the included studies, which could impact the generalizability of the results. The analysis is based on existing literature up to the cutoff date and may not capture more recent developments in the field. The risk factors to CRE were not presented for all the studies that qualified to the meta-analysis which makes it difficult to establish control measures. Publication bias could affect the prevalence estimates, as studies with significant findings are more likely to be published.

### Implications for public health

The findings of this study have important implications for public health. The high prevalence of *Enterobacteriaceae* infections, especially in hospitals, highlights the need for infection prevention and control measures. These measures should include hand hygiene, proper cleaning and disinfection of surfaces, and the use of antibiotics only when necessary.

The increasing prevalence of Carbapenem-resistant *Enterobacteriaceae* is a serious threat to public health. These bacteria can be difficult to treat, and they can cause serious infections that can lead to death. It is important to continue research on new antibiotics and other treatments for *Enterobacteriaceae* infections.

There is need to improve article scrutiny during review to allow better information generation that could potentially lead to control of the MDR bacteria isolates.

## Conclusion

In conclusion, this systematic review and meta-analysis highlight the substantial prevalence (43.06% (95% CI 21.57–66.03)) of *Enterobacteriaceae* infections, the variability in prevalence based on the source of isolation, and the concerning emergence of carbapenem-resistant strains, particularly within *Klebsiella pneumoniae,* particularly the *bla*KPC production. Monitoring and surveillance programs should consider the presence of the *bla*KPC-2 gene in environmental samples, as these reservoirs can contribute to the spread of resistance determinants among both clinical and non-clinical settings. These findings emphasize the importance of ongoing research, surveillance, and infection control measures to address the public health challenges posed by *Enterobacteriaceae* infections and antimicrobial resistance. Additionally, further studies are needed to explore the mechanisms driving the predominance of specific bacterial species and the distribution of resistance genes within this bacterial family.

### Recommendations

In clinical practice, the utilization of carbapenems should strictly follow resistance testing methods such as both DST and PCR. Additionally, when administering carbapenems to individuals, it is recommended to combine them with carbapenemase inhibitors as a proactive measure. It is crucial to conduct comprehensive large-scale studies focusing on the prevalence of *bla*KPC, *bla*NDM, *bla*OXA-48, *bla*IMP and *bla*VIM in *Enterobacteriaceae* to comprehend the spectrum of resistant genes in both community and healthcare environments.

## Data Availability

Data is available with journal author name estimator (JANE), PubMed, Google scholar and African Journals Online (AJOL).
